# Medical-Grade PCL Based Polyurethane System for FDM 3D Printing—Characterization and Fabrication

**DOI:** 10.3390/ma12060887

**Published:** 2019-03-16

**Authors:** Agnieszka Haryńska, Justyna Kucinska-Lipka, Agnieszka Sulowska, Iga Gubanska, Marcin Kostrzewa, Helena Janik

**Affiliations:** 1 Department of Polymer Technology, Faculty of Chemistry, Gdansk University of Technology, 80-232 Gdansk, Poland; agnieszka.harynska@pg.edu.pl (A.H.); agnsulow@student.pg.edu.pl (A.S.); igaguban@pg.edu.pl (I.G.); heljanik@pg.edu.pl (H.J.); 2 Department of Organic Materials Technology, Technical University of Radom, 26-600 Radom, Poland; m.kostrzewa@uthrad.pl

**Keywords:** PCL based TPU, material characterization, fused-filament fabrication, fused deposition modeling, 3D printing, polyurethane filament

## Abstract

The widespread use of three-dimensional (3D) printing technologies in medicine has contributed to the increased demand for 3D printing materials. In addition, new printing materials that are appearing in the industry do not provide a detailed material characterization. In this paper, we present the synthesis and characterization of polycaprolactone (PCL) based medical-grade thermoplastic polyurethanes, which are suitable for forming in a filament that is dedicated to Fused Deposition Modeling 3D (FDM 3D)printers. For this purpose, we synthesized polyurethane that is based on PCL and 1,6-hexamethylene diisocyanate (HDI) with a different isocyanate index NCO:OH (0.9:1, 1.1:1). Particular characteristics of synthesized materials included, structural properties (FTIR, Raman), thermal (differential scanning calorimetry (DSC), thermogravimetric analysis (TGA)), mechanical and surfaces (contact angle) properties. Moreover, pre-biological tests in vitro and degradation studies were also performed. On the basis of the conducted tests, a material with more desirable properties S-TPU(PCL)0.9 was selected and the optimization of filament forming via melt-extrusion process was described. The initial biological test showed the biocompatibility of synthesized S-TPU(PCL)0.9 with respect to C2C12 cells. It was noticed that the process of thermoplastic polyurethanes (TPU) filaments forming by extrusion was significantly influenced by the appropriate ratio between the temperature profile, rotation speed, and dosage ratio.

## 1. Introduction

Global interest in three-dimensional (3D) printable biopolymers for applications, such as drug delivery devices, scaffolds in tissue engineering, as well as artificial organs for surgery trainings, are constantly growing [1,2,3]. While there are more and more modifications of 3D printers for medical purposes [4,5,6], new material solutions are also needed.

Synthetic polymers, including polylactonesthat were obtained from L- or DL-lactide monomers, glycolide, ε-caprolactone, or p-dioxanone have found wide clinical application [7,8]. Parts of polylactones, especially those with a high content of L-lactide or glycolide, are materials with increased stiffness. For implanted parts in the areas of soft body tissues, such as the skin [9], adipose tissue [10], cardiovascular area [11], or for anatomical models [12], more soft and elastic biopolymers are needed than rigid ones. There is a constant need for biomaterials that can be modified both in terms of biodegradability and mechanical properties. In this context, polyurethanes are a very promising group of synthetic polymers.

Thermoplastic polyurethanes (TPUs) consist of alternating rigid (hard) and flexible (soft) blocks, which, under appropriate conditions, can undergo the separation phenomenon of the blocks, leading to nanodomain morphology, which acts as a physical crosslinking of the system and the materials, called thermoplastic elastomers (TPEs), are then created. TPEs behave like elastomers, but they can be processed like thermoplastic materials. The latter properties are very important in making filaments from TPU. Flexible segments consist of the reaction products between polyols (polyesters, polyethers, or polycarbonates) and diisocyanates, while rigid segments are the result of the reaction between diisocyanates and small molecular chain extenders [13]. The flexible segments provide elastomeric character, while rigid segments usually provide additional strength due to hydrogen bonding between urethane groups [14]. The use of different starting substrates, change of their molecular mass, and the ratio of rigid and flexible segments may lead to polyurethanes with different physical and physicochemical properties, or biodegradability, tailored for the intended use [15]. Moreover, properly designed thermoplastic polyurethanes (TPUs) are suitable materials for use in 3D printing by the Fused Deposition Modeling (FDM) method [16,17]. FDM uses materials that are based on a thermoplastic polymer matrix in the form of a thin filament of a given diameter. Using computer software and 3D models with the STL. Format, the FDM printer creates ready-made 3D structures layer by layer.

Polycaprolactone (PCL) is a one of the most commonly used biopolymers in medical application. Ease of processing and high flexibility characterizes PCL. It is also a material that is thoroughly examined by scientists in the context of biological interactions in vivo and in vitro, thanks to which the FDA approves many medical devices that are based on PCL polymer. Due to these features, PCL is successfully used as a polyol in the synthesis of medical polyester urethanes (Table 1).

Taking into account the literature overview on medical grade TPUs, which is shortly presented in Table 1, we found that there are only a few systems of PCL/based TPUs that are dedicated as filaments for 3D printing technology. In most of the presented works (Table 1), the obtained polyester-urethanes for medical application were processed by electrospining or hydrogels were formed or thin films were produced. Guney et al. [24] paper is one of rare examples, in which the synthesis, characterization, and formation of a medical filament for 3D printing in FDM technology are presented. They synthesized thermoplastic tough polyurethane hydrogels from triblock copolymers (PCL-PEG-PCL) and HDI diisocyanate. They described the synthesis, characterization, and formation of filament by the melt-extrusion process. They also successfully tested the filament on a 3D FDM printer. Another paper in which new 3D FDM filaments were obtained with the use of holt-melt extrusion is that one of Fuenmayor et al. [25]. Designed filaments that were based on PCL and PVP were dedicated to solid dosage forms. Researchers characterized the process and problems occurring during the formation of filaments for FDM 3D printing via melt-extrusion.

In our previous work [16], we examined the effect of TPU filament formation on its selected properties. The results showed no significant changes on the chosen properties of the obtained filament after the melt-extrusion extrusion process. Notwithstanding, the final characteristic of the printed 3D object (mechanical characteristic [26,27] or biological properties [28,29]) also depends on the 3D printing parameters and the design of 3D object. Therefore, we decided to prepare polyurethane filament for the use in Fused Filament Fabrication 3D printing with potential to be used as a medical-grade material and characterize solid material before its formation into filament. 

## 2. Materials and Methods

In order to receive the intended results, the following steps were taken:

- Synthesis of aliphatic polyester-urethane S-TPU(PCL) with PCL diol as a polyol,
- Material (S-TPU(PCL) characterization, which included:
  ○ structure studies (FTIR, Raman);
  ○ mechanical properties (static tensile test, hardness);
  ○ thermal characterization(differential scanning calorimetry (DSC), thermogravimetric analysis (TGA));
  ○ surface properties (surface free energy, contact angle);
  ○ interaction with media (short-, and long-term degradation tests, water absorption test); and,
  ○ initial biological test (cytotoxicity).
- Formation of filament F-TPU(PCL) for FDM 3D printing via melt-extrusion process

### 2.1. S-TPU(PCL) Synthesis

In accordance with previous works [16,30] a two-stage (prepolymer method) synthesis of polyester urethanes (S-TPU(PCL)) was carried out. The first stage of the synthesis was the reaction of the polyol (PCL diol), with an excess of diisocyanate (HDI) resulting in a prepolymer mixture containing an isocyanate terminated prepolymer (building soft segments) and an excess of unreacted diisocyanate. The next step was the chain extension reaction, by adding a small molecular weight chain extender (BDO) to the prepolymer mixture. The description of raw materials that were used in polyurethane synthesis is described in Table 2. No catalysts were used for the synthesis of polymeric materials, which seems reasonable for medical application. What is more, the lack of catalyst can help to improve the biocompatibility of materials. Tanzi et al. [31] demonstrated that the catalysts commonly used in the synthesis of polyurethanes (such as tin octoate, dibutyltindilaurate, 1,4-diazabicyclooctane, tetramethylbutanediamine) increase the cytotoxicity of the polymer with respect to human endothelial cells. Hence, the synthesis of polyurethanes for medical applications are more often carried out without the use of the above catalysts [19,32].

Before the synthesis, PCL, diol, and BDO were dried in a vacuum for four hours at 100 °C, while stirring. HDI was used without prior treatment. The dried PCL diol, was placed in the reactor and then heated to melt. After reaching a temperature of 40 °C, HDI was added in portions (to obtain a prepolymerwith an 8% excess of unbound isocyanate groups -NCO). After the prepolymerization stage was carried out at 80 °C for 3 h, the reaction mixture was left for 12 h at room temperature to complete the reaction. Subsequently, the chain extension process was conducted by adding the BDO to preheated (60 °C) prepolymer mixture (mechanical stirring, 3 min, 1050 rpm). The chain extender was added in two different molar ratio of NCO groups to OH groups equal to 0.9:1 and 1.1:1, respectively. After that, the mixture was degassed and then poured into a preheated mould (100 °C). Finally, the material in the mold was put into the dryer for 24 h.

### 2.2. S-TPU(PCL) Characterization

The chemical structure of samples was tested using an FTIR Nicolet 8700 (Thermo Fisher Scientific, Waltham, MA, USA) spectrometer at room temperature. The equipment included a Specac’s Golden Gate module and a single reflective ATR (Attenuated Total Reflectance) diamond, used to attenuate the total infrared reflection. The range of recorded spectra was 4000–500 cm^−1^. Each sample was scanned 254 times at a resolution of 4 cm^−1^. Additionally, the chosen samples from short-term degradation test were tested with FTIR.

Raman spectroscopy was also applied to study a chemical composition of the sample. It uses monochromatic light and its phenomenon of inelastic scattering with molecules. Measurements were performed using micro-Raman system (InVia, Renishaw, Wotton-under-Edge, UK). The spectra were collected at randomly selected locations (50x magnification) over the 100–3200 cm^-1^ range, at room temperature. The used wavelength was of 785 nm (red laser) and the power of laser was of 15 mW. Each sample was scanned five times.

The hardness of the samples was measured using the Shore type (A and D) electronic hardness tester (Zwick/Roell), in accordance with the PN-EN ISO 868:2004 standard. Flat samples of solid polymer materials with a thickness of about 8 mm were used. The results were averaged from 10 measurements. A density measurement of samples was carried out using the analytical balance (AS 110/C/2, Radwag, Radom, Poland) equipped with a density set. The mass of samples was determined in the air and then in water. The results were the arithmetic mean of eight measurements. The static tensile strength test of the samples was conducted using Zwick/Roell Z020 table resistance machine (Wrocław, Poland) integrated with the testXpert^®^ I program in accordance with the PN-EN ISO 527-2:2012 standard (dumbbell shaped samples). The test was conducted at room temperature with a crosshead speed of 500 mm/min and initial force of 1 N. The results are the arithmetic mean of eight measurements.

Differential scanning calorimetry (DSC) measurements were conducted using a heat-fluxNetzsch DSC 204F1machine (Netzsch, Selb, Germany). The thermal properties of samples with the mass of 7–8 mg were investigated at the temperature range of −85 °C to 220 °C (heating rate 10 °C/min, cooling rate 20 °C/min), under nitrogen atmosphere (N_2_ flow rate 20 mL/min).

The thermal stability of the obtained polyurethanes was tested with thermogravimetric analysis (TGA) using a Q1000 TAInstruments (New Castle, DE, USA). Samples of 3–6 mg mass were heated under a nitrogen atmosphere from 20 °C to 700 °C, with a heating rate of 10 °C/min.

To determine the wettability of the surface layer and surface free energy (SFE) of synthesized polyurethanes, the contact angle test by the sessile drop technique was carried out. The analysis was performed while using Kruss Goniometer G10 (KRÜSS GmbH, Hamburg, Germany) with drop shape analysis software DSA4.1 (5.5.3 version). On purified with n-hexane samples surface, a 2 µL droplet of liquid was placed and the images were taken. The SFE was determined by two methods, the Owens-Wendt method and Acid-base approach. The first method requires the use of polar and dispersion liquids, therefore the distilled water and diiodomethanewere used. In turn, acid-base method needs the use of three polar liquids (formamide, ethylene glycol, water) and one dispersive/non-polar (diiodomethane) to calculate the SFE. The results of contact angle for each of the four liquids were an average of six measurements taken on randomly selected surface points.

### 2.3. Cytocompatibility (In Vitro)

The cytotoxicity evaluation was performed according to the ISO 10993-5:2009 standard. The cell metabolic activity was estimated by using MTT assay. Extract: prior the extract preparation, polyesterurethanes were sterilized with ice-cold 70% EtOH overnight and then exposed to UV for 15 min at each side. The sterile samples of S-TPU(PCL)0.9/1.1 were incubated for 24 h in culture medium: Dulbecco’s Modified Eagle’s Medium (High glucose DMEM, Sigma Aldrich, Poznań Poland) containing 10% Fetal Bovine Serum (FBS, HyClone, Pittsburgh, PA, USA) and 1% penicillin/streptomycin (P/S, Sigma Aldrich, Poznań, Poland). After this, the time extracts were added to the cells. Cell culture: C2C12 murine myoblasts (ATCC) were expanded and maintained in DMEM, containing 10% FBS and 1% P/S. C2C12 myoblasts were seeded at a cell density of 10^5^ cells per well in a 24-well tissue culture plate (BD) and then incubated. After 24 h in culture, the media was changed, and the 100% extract of polyester urethanes was added. Following 72 h incubation, the experimental materials were removed and the cytotoxicity was assessed by MTT assay kit (Sigma Aldrich, Poznań, Poland). A plate reader was used to measure the conversion of the tetrazolium salt to its colorimetric indicator at a wavelength of 490 nm.

The statistical analysis was performed with the use of the Origin Pro 8.5 (Washington, DC, USA). To evaluate statistical differences, the two-way ANOVA (α = 0.05) and post hock Tukey test (α = 0.05) were used.

### 2.4. Degradation Study and Water Absorption Test

Standard, from medical point of view [3], degradation tests of prepared polyurethanes were conducted to estimate the interaction of polymer with different media. For this purpose, long-term degradation studies (84 days, 37 °C) in phosphate buffer (PBS) and short-term degradation (32 days, 37 °C) in 2M HCl and 5M NaOH were performed [3,33]. Round samples (diameter 7 mm) were cut before testing and then dried to a constant weight (4 h, 60 °C). Six samples were taken for each of the tests. The samples were placed in 3 mL wells test plate, immersed with 2 mL of the appropriate solution (PBS, HCl, or NaOH), and then incubated at 37 °C. During the short-term degradation test (HCl/NaOH), sample’s weight loss was measured after one, two, three weeks, and 32 days. In turn, for the long-term degradation process (PBS), the weight of the samples was measured afterone week, one month, and three months. After specified time intervals, the samples were rinsed several times in distilled water, then dried (three days, 37 °C), and weighed again using an analytical balance (Radwag, Poland). The degree of degradation was determined by the percentage loss of mass of the sample over time. Additionally, after short-term degradation (in 2 M HCl and 5 M NaOH), the samples were observed under the optical microscope (Bresser, Rhede, Germany) camera integrated with VidCap 5.1 program (Microsoft, Washington, DC, USA), mag. 40x and 400x).

The water absorption (WA) test was carried out on circular samples with a diameter of 7mm. The dried samples (4 h, 60 °C) were weighed and placed in the wells of the test plate, and then flooded with 2 mL of distilled water. The incubation time of samples was as follows: 0.5 h; 1 h; 5 h; 24 h; 48 h; and, 72 h. After removing the samples from the test plates, the excess of water was gently removed with tissue paper, and the samples were re-weighed. Water absorption was calculated based on the amount of water absorbed according to Equation (1). The obtained results constituted the arithmetic mean of four measurements.

(1) $$WA=\frac{m_{i}-m_{0}}{m_{0}}\cdot 100\%$$

where: *WA*—water absorption [%], *m*_0_—initial mass of dry sample [g], and *m_i_*—mass of sample after incubation in water [g].

### 2.5. Filament Formation

Synthesized bulk S-TPU was granulated while using high-speed mill (WittmannBattenfeld, Grodzisk Mazowiecki, Poland) and dried in laboratory oven (60 °C, 24 h) before the melt-extrusion process. Single screw extruder (Brabender, Duisburg, Germany) with two heating zones was used toconvert the synthesized polyurethane (S-TPU) into polyurethane filament (F-TPU). The custom-made molding nozzle diameter was equal to 1.9 mm. The processing parameters, like temperature profile, dosage rate, and rotation speed were tested to obtain a dimensionally stable extrudate. Electronic caliper was used to control the filament diameter.

## 3. Results and Discussion

### 3.1. Material Characterization S-TPU(PCL)

#### Chemical Characterization (FTIR, Raman)

**Figure 1:**
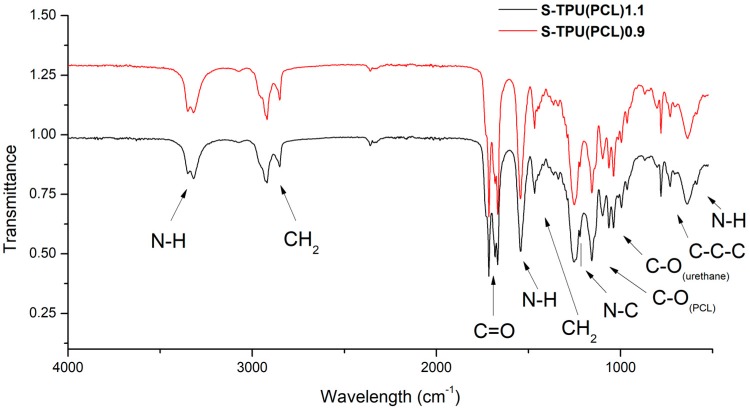
FTIR spectra of synthesized S-TPU(PCL)0.9/1.1.

Based on the FTIR spectra (Figure 1), the chemical structure of the synthesized materials was analyzed. Both FTIR spectra were similar. The presence of the main peaks that are characteristic of polyurethanes was observed, which confirmed the positive reaction of polyester urethanes synthesis. The absorption bands at 3318 cm^−1^, 1686–1660 cm^−1^, and 1223 cm^−1^ described the vibrations of amide bonds, N–H, C=O, and C–N, respectively, suggesting the formation of urethane bonds. The characteristic peaks at 2917 cm^−1^ and 2850 cm^−1^ corresponded to the asymmetric and symmetric vibrations of the –CH2– groups. Two strong and high intensity peaks at 1717 cm^−1^ and 1160 cm^−1^ were the stretching vibrations of C=O and C–O bonds of the ester groups of the soft PCL segment. Table 3 presenteda detailed description of the FTIR spectra. No intensive peak with a wavenumber around 2280 cm^−1^ indicated the complete reaction of isocyanate groups (lack of –NCO groups).

**Figure 2:**
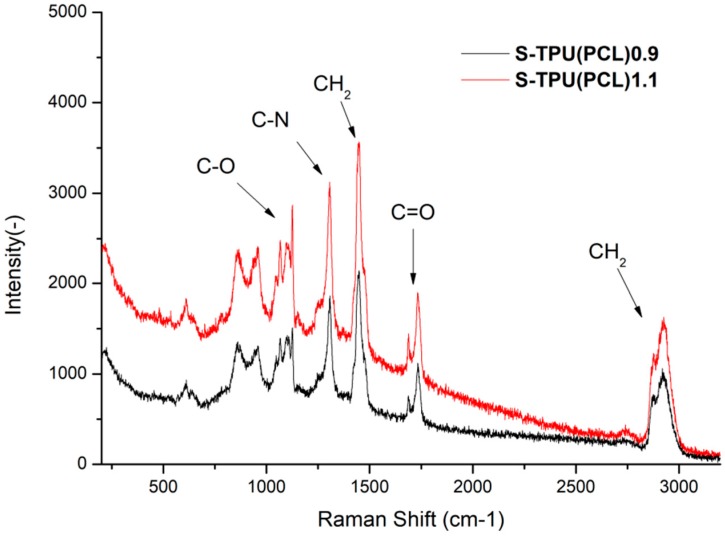
Raman spectroscopy of synthesized S-TPU(PCL)0.9/1.1.

Raman spectroscopy was performed to complete the FTIR study (Figure 2). The results proved to be complementary. Thus, it confirmed the presence of hydrogen bonded C=O band in the structure of synthesized polyester urethanes [33]. However, it seems thata more complementary technique to describe polyurethanes structure is the FTIR study. A visible change between samples intensity of the Raman spectrum was noted. This is related to the phenomenon of fluorescence of the studied materials. It can be seen that S-TPU(PCL)1.1 exhibited a noticeably higher degree of illumination as a result of interaction with the laser light (785 nm). The obtained Raman spectrum was complementary and it confirmed the FTIR studies well.

#### Thermal Characterization (DSC, TGA)

**Figure 3:**
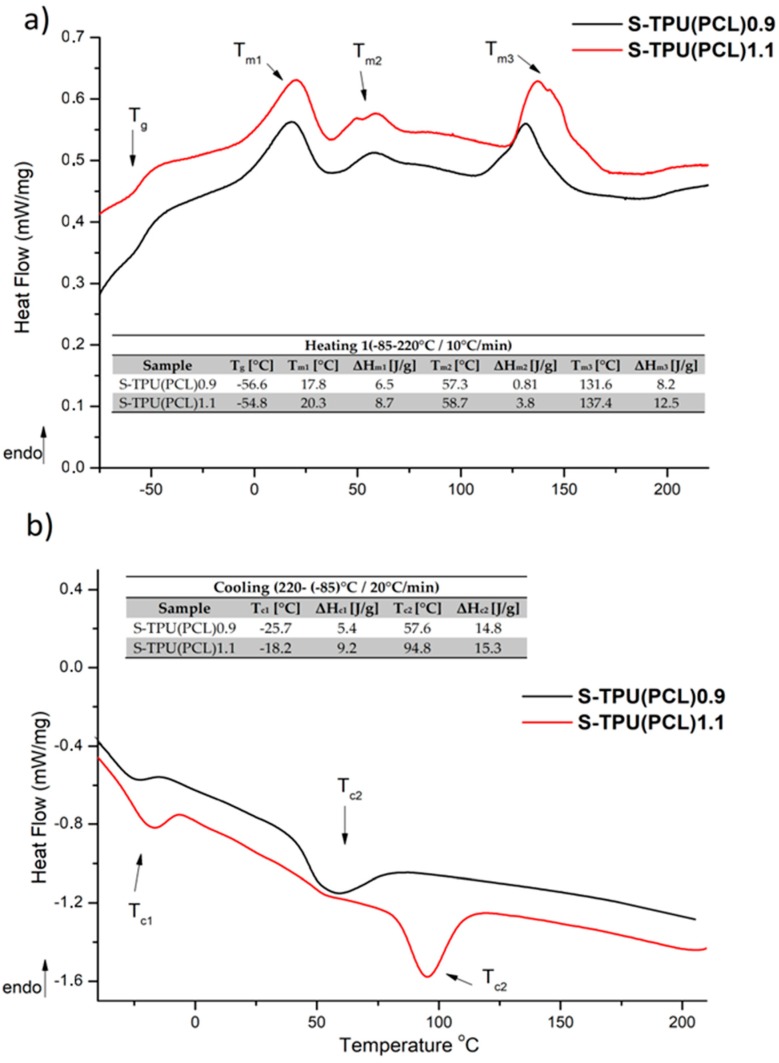
Differential scanning calorimetry (DSC) curves of S-TPU(PCL)0.9/1.1 with designated values; (**a**) first heating run, (**b**) first cooling run.

Figure 3 presents the results of DSC study. The measurements were used to determine the glass transition temperature (T_g_), melting temperature (T_m_), and heat enthalpy (ΔH_m_). The first heating run showed the glass transition (T_g_) at the temperature of −54.8 °C for S-TPU(PCL)1.1 and around −56.5 °C for S-TPU(PCL)0.9. This corresponds to the glass transition temperature of soft segments having PCL in their structure. The glass transition temperature for neat PCL, according to the literature, is around −60 °C [34]. Moreover, first heating run showed three melting temperatures. The first melting point was observed in the range of around 20 °C and the second ones at around 57 °C. They can both be attributed to the melting of soft segments, in which homo and heterogenous nucleation, according to the literature, can take place for neat PCL [35,36,37]. The third melting point with the highest heat enthalpy occurred at over 130 °C. This endothermictransition is due to the melting of strong hydrogen bonded chains of hard segments in both materials (T_m3_ = 131 °C, ΔH_m_ = 8.2 J/g for S-TPU(PCL)0.9 and T_m3_ = 137 °C, ΔH_m_ = 12.5 J/g for S-TPU(PCL)1.1). In turn, the first cooling run showed crystallization temperatures of soft segments (T_c1_) and hard segments (T_c2_). The crystallization of both regions in S-TPU(PCL)1.1 occurred at noticeablyhigher temperatures than in the case of S-TPU(PCL)0.9. 

**Figure 4:**
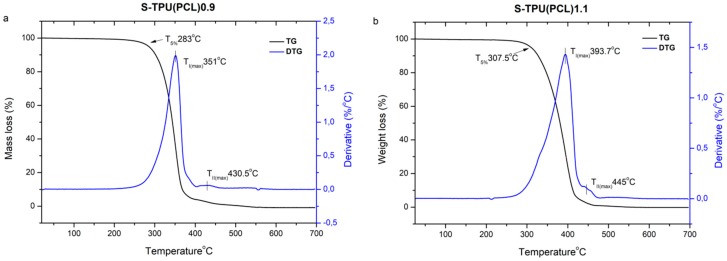
TGA graph of S-TPU(PCL)0.9/1.1 with a derivative curve (DTG).

Figure 4 and Table 4 present the results of the thermogravimetricanalysis. The thermal stability of both synthesized polyester urethanes is above 260 °C (which shows that they can be processed for filaments until up that temperature), whilethe complete degradation occurs between 455–490 °C. Both thermo grams of the obtained materials have a similar shape. The determined derivative curves (DTG) (Figure 4) allowed forobservinga two-stage decomposition process that might indicate the presence of micro-phase separation in synthesized polyester-urethanes [38]. Thermal degradation begins with the thermal dissociation of urethane bonds (the weakest against the temperature) and it occurs at 351 °C for S-TPU(PCL)0.9 and at 393 °C for S-TPU(PCL)1.1(I stage of decomposition, T_maxI_). The observedsmall peak of the second stage of decomposition (T_maxII_)is related to the thermal decomposition of soft segments that are present in polyesterurethanes (PCL part of chain). The results in Table 4 indicate adifferent path of degradation S-TPU(PCL)1.1 and S-TPU(PCL)0.9. It can be explained by the possibility of creating some cross-linking for the sample with 1.1:1 isocyanate index. For comparison, the decomposition temperatures of PCL tested by [39] is around 350 °C.

#### Physico-Mechanical Poperties

Table 5 presents the results of hardness, density, and static tensile tests of the synthesized polyuretane materials. The density value was slightly different for both of the samples and equaled to 1.118 g/cm^3^ for S-TPU(PCL) 0.9 and 1.021 g/cm^3^ for S-TPU(PCL)1.1, respectively. However, significant differences in strength properties between materials were noticed. The static tensile test showed that S-TPU(PCL)1.1 had almost three times higher tensile strength (~21 MPa) and elongation at break (~720%) than S-TPU(PCL)0.9 (~8MPa, ~200%), which was closely related with changes in structure of both materials. Isocyanate index NCO:OH=1.1:1 in S-TPU(PCL)1.1 might have contributed to the partial cross-linking of polymer chains, which is manifested by better mechanical properties, significantly higher elongation at break, and higher (~91°ShA) hardness than S-TPU(PCL)0.9 sample (~84°ShA). These observations were consistent with the research that was carried out by Kasprzyk et al. [40], in which, among others, the influence of the isocyanate index on selected mechanical properties of polyesterurethanes was examined.

The mechanical properties of polyurethanes depend among others on: degree of crystallinity, concentration and structure of rigid segments, or ability of soft segments to crystallize [41]. By changing the weight ratio of hard and soft segments, the mechanical properties of polyurethanes can be modified. With an increasing ratio of NCO groups to OH (0.9 to 1.1), the tensile strength increased and then reached a maximum value of 21.4 MPa for S-TPU(PCL)1.1.

#### Surface Properties (Contact Angle and Surface Free Energy)

Table 6 and Table 7 present the results of study on surfaces properties of S-TPU(PCL)0.9/1.1. The obtained materials had water contact angle in the range from 104° (S-TPU(PCL)0.9) and 107° (S-TPU(PCL)1.1). The contacts anglesthat were studied in diiodomethanewere the lowest, ~63° and 59° for S-TP(PCL)0.9 and S-TPU(PCL)1.1, respectively. In turn, the values of contact angle that were obtained using formamide and ethylene glycol were similar and were around 81–88° (S-TPU(PCL)0.9) and 83–90° (S-TPU(PCL)1.1).

Contact angle and surface energy are important parameters, especially inmaterials that are used in biomedicine. These parameters affect the interaction of the material with the cells and allow them to adhere. The common definition says that biocompatible polymers should have a water contact angle between (55–75°), which ensures adequate adhesion of the cells to the substrate [42]. However, according to Menzies et al. [43], low values of contact angle do not provide biocompatible properties of the tested polymer. In some medical applications (e.g., dental implants), the hydrophobicity of the surface is favored to prevent excessive intervention and adhesion of live cells or organs (bacteria) that can cause the erosion of the polymeric material.

An increase in the isocyanate index (up to 1.1) resulted in an increase in contact angles towards polar liquids, and thus the total surface energy of S-TPU(PCL)1.1 (~23 and 27 mN/m) was lower than for S-TPU(PCL)0.9 (~25 and 28 mN/m), as calculated by Owens–Wendt and acid–base theory, respectively. It should be point out that too high surface energy (high hydrophilicity of the surface) can even causedisruption ofcell-cell interactions and reduce biocompatibility [43]. Therefore, depending on the application, biomaterials with both hydrophilic and hydrophobic properties should be developed.

#### Interactions with Media (Water Absorption Test, Short-, and Long-Term Degradation Tests)

**Figure 5:**
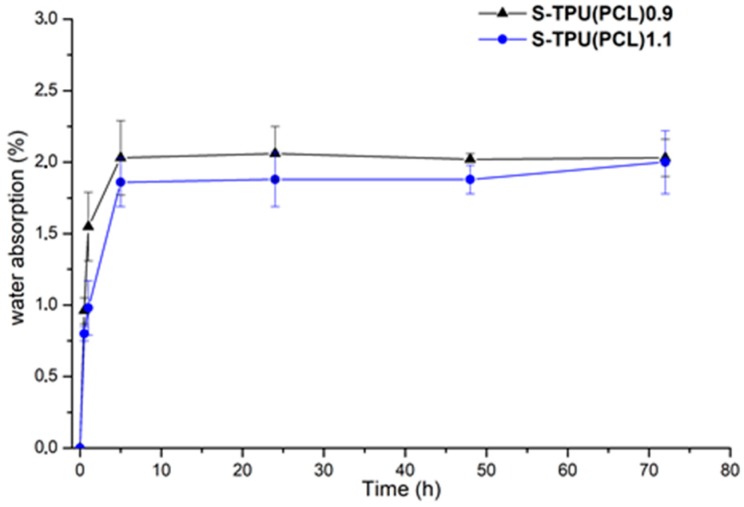
Graph of percentage water absorption change over time of S-TPU(PCL)0.9/1.1 samples.

Figure 5 summarizes the results of absorption test of S-TPU(PCL)0.9 and 1.1 samples during incubation in distilled water. These data show the percentage of water that was absorbed by polyester-urethane materials during the measurement. At the first hour of incubation, the largest increase in both samples mass was observed. After this time, the rate of water absorption remained at a relatively constant level (between 1.8–2.1%). After 72 hours, the water absorption of both samples was approximately 2.2%. 

Data from the graph showed that the S-TPU(PCL)0.9 sample wasslightly more susceptible to water absorption in the initial stage of the study. These observations coincided with the results of the water contact angle measurements: S-TPU (PCL)0.9 (~104°), S-TPU (PCL)1.1 (~107°), therefore less hydrophobic material absorbs more water. Slower water absorption of S-TPU(PCL)1.1 might also be connected with difficult access of water molecules between partially cross-linked chains.

Table 8 summarizes the results of a sample incubation test in phosphate buffer (PBS) at 37 °C. PBS buffer (pH~7.4) provides a hydrolytic degradation environment. After one week of incubation, a very slight weight loss was observed. However, after 12 weeks of incubation, the mass of the samples increased slightly, which could be caused by the precipitation of salt on the samples surface or bywater absorption. There was no significant weight loss after 12 weeks of incubation, thus the polyester urethanesthat were tested in this work proved to be stable during long-term degradation in the PBS environment.

**Figure 6:**
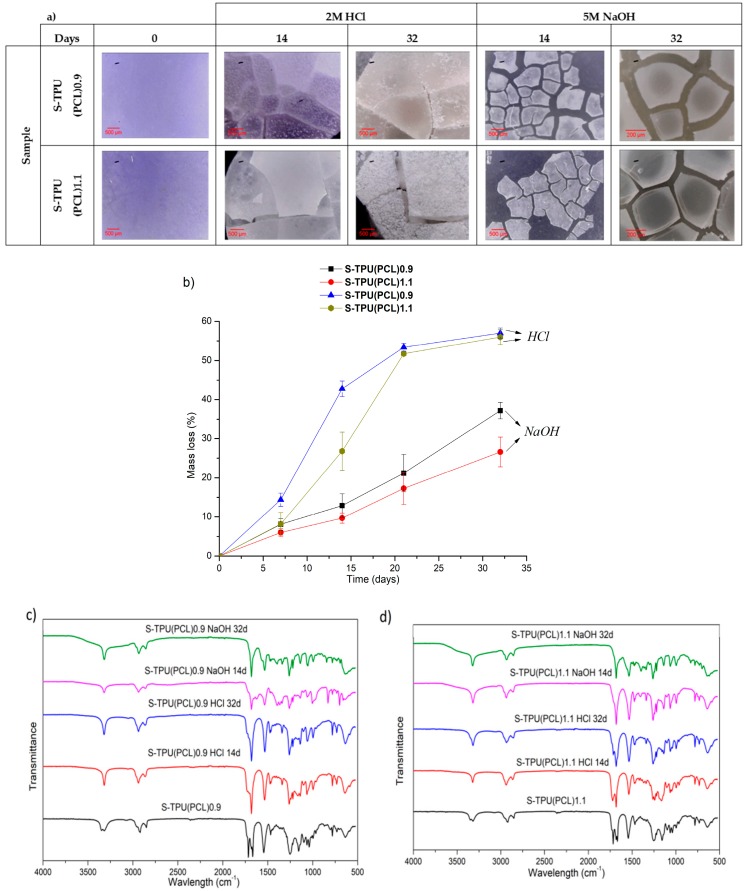
Results of study on short-term degradation in 2M HCl and 5M NaOH of S-TPU(PCL)0.9/1.1, (**a**) optical microscopy, (**b**) graph of percentage mass loss during degradation process, (**c**,**d**) FTIR spectrum measured at different time of degradation of S-TPU(PCL)0.9.

Short-term degradation (in concentrated aqueous solutions of 2 M HCl and 5 M NaOH) included microscopic studies of the degradation progress (Figure 6a), measurements of the percentage loss of mass of the tested samples (Figure 6b), and FTIR spectra analysis from degradation progress (Figure 6c,d). The accelerated degradation results clearly illustrate thatthe degradation of polyester-urethanes was highly dependent on the environment in which the process was carried out. The tested materials were more susceptible to degradation in an acidic than the basic environment. In 2 M HCl, the mass of samples after 32 days decreased by 57 and 56%, respectively, for S-TPU(PCL)0.9 and S-TPU(PCL)1.1. After the same time, the mass of samples in 5 M NaOH decreased by 37% and 26%, respectively, for S-TPU(PCL)0.9 and S-TPU(PCL)1.1.

Microscopic photos showed that, after 32 days of incubation in the acidic environment, the structure of both samples was destroyed, while the erosion of the material in the alkaline medium progressed surface (the material did not disintegrate by volume, Figure 6a). It was also noted that, in the initial stages of incubation in both media, the samples with an isocyanate index NCO: OH 1.1:1 were more resistant to erosion (14 days incubation in HCl, S-TPU(PCL)1.1 weight loss equal to 26%, when for S-TPU(PCL)0.9 was 42%). The mechanism and degradation progress of synthesized materials werealso observed by FTIR spectra analysis (Figure 6c,d). The obtained FTIR spectra showed that, during incubation in aqueous HCl and NaOH solutions, the band at 1720 cm^−1^, corresponding to the stretching vibration of carbonyl group (PCL part), disappeared (Figure 6c,d). However, it should be noted that this effect was more noticeable in the case of the alkaline environment and the sample with a lower isocyanate index (NCO: OH = 0.9:1). It can be seen that erosion at the beginning of the test occurred through the destruction of ester bonds in soft segments and the degradation of hydrogen bonded urethane bonds (visible disappearance of the N–H stretching vibration around 3340 cm^−1^). The intensity of the peak originating from N–H deformation (~1542 cm^−1^) decreased, which suggested the breakdown of urethane bonds. Peaks originating from νC–O occurring in the urethane bond (at 1065, 1038 cm^−1^) were also deformed. An appearance of peak around 1220–1200 cm^−1^ might be associated with C–O stretching vibration that originated from alcohols formed as a result of urethane bond degradation.

A rapid rate of degradation of polyester-urethanes (both S-TPU(PCL)0.9 and S-TPU(PCL)1.1) can be observed in the acidic environment after five days, until about 21 days. After 21 days, the degradation rate remained relatively stable. In turn, a completely different rate of mass changes over time, as compared to degradation in the acid environment, characterizes the degradation in the alkaline environment. In 5 M aqueous NaOH solution, the rate of degradation had a relatively linear character. The hydrolysis of polyesters depends on the pH of the environment. The environment containing the acidic proton is significantly more aggressive to the obtained polyester-urethanes. A similar course of short-term degradation behind concentrated aqueous solutions was observed in our earlier works [33].

#### Cytotoxicity (In Vitro)

**Figure 7:**
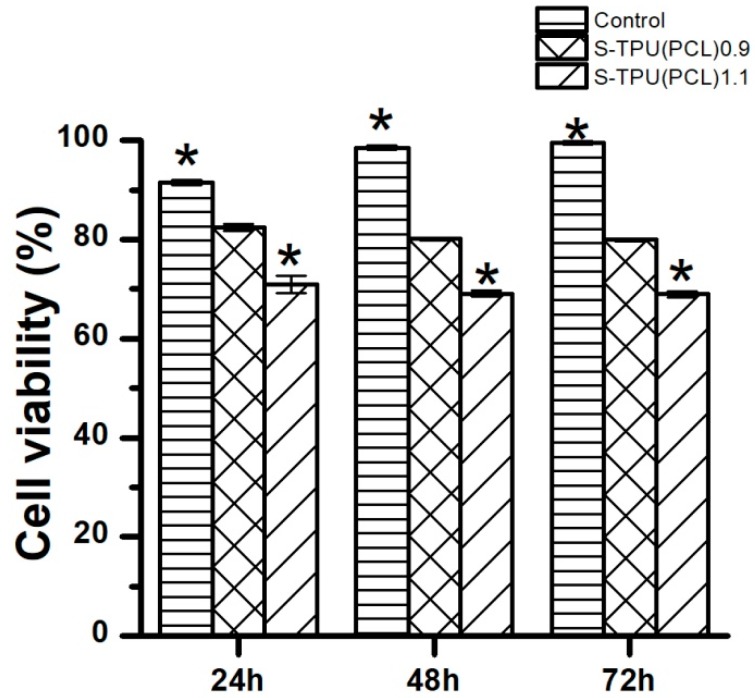
The effect of S-TPU(PCL)0.9/1.1 extracts on the growth of C2C12 cells tested via MTT assay after 24, 48, and 72 h of incubation.

Figure 7 presents the effect of synthesized materials extracts on the growth of C2C12 cells that were studied by MTT assay. The cytotoxicity examination is one of the tests to determine whetherthe material is biocompatible and suitable for medical application.

Figure 7 shows that C2C12 cells viability was above 80% for the obtained S-TPU(PCL)0.9, and this difference was not statistically significant (*p* < 0.05) in comparison to the control, which represents 100% of cell viability. On the other hand, S-TPU(PCL)1.1 represents lower cell viability (~70%) in comparison to the S-TPU(PCL)0.9, but the results were not statistically different between each other. The statistical difference was observed between S-TPU(PCL)1.1 and the control, which means that the biocompatibility significantly decreased for S-TPU(PCL).1.1 (*p* < 0.05). The cytotoxicity test showed that S-TPU(PCL)0.9 that was obtained with medical-grade PCL macrodiol may be suitable for medical applications of such filaments.

### 3.2. Melt-Extrusion of F-TPU(PCL)0.9 Filament

It was necessary to choose the appropriate temperature profile, extrusion speed, and the degree of granulate dosage in order to obtain a stable dimensional filament. Table 9 presents the results of the melt-extrusion process. In process number 1, the temperature profile between 165–175 °C was too low and the inclusions in the material causing discontinuity of the filament were observed. It was a non-plasticized polymer granulate, therefore, in process 2, both the temperature and the extrusion speed were increased in order to increase the pressure in the extruder barrel, which could improve the degree of plasticization. It was noted that the changes introduced led to degradation of the material, the temperature turned out to be too high, and a material with many visible blisters of a yellowing color was obtained. In process 3, the temperature was again lowered, and plasticized material was obtained, however with too large diameter (too much material in the cylinder and the insufficient temperature of the zone 2 (T2) caused a large swelling of the extruded wire). The above observations allowed for the selection of appropriate parameters, which ensured obtaining a stable filament F-TPU(PCL)0.9 (process 4).

## 4. Conclusions

The purpose of this work in the first step was the synthesis and detailed characterization of polyester-urethanes that are based on biodegradable PCL and aliphatic HDI, differing by the isocyanateindex (NCO:OH = 0.9: 1 and 1.1:1, respectively) and in the second step processing of thus obtained TPU into filament for use in 3D FDM printers. On the basis of the mechanical, physic-chemical, and thermal characteristics, it was found that, with the increase of NCO:OH index, the Shore hardness, tensile strength, and thermal stability of solid polyurethanes increased. With the increase of the NCO:OH ratio, the water absorption decreases, which is adequate to the result of contact angle studies and the higher hydrophobicity of the S-TPU(PCL)1.1 sample. The above relationships were probably due to the partial cross-linking of S-TPU(PCL)1.1 polymer chains. Both of the materials were stable in the PBS environment. On the basis of the conducted short-term degradation study, it was observed that the obtained materials were more susceptible to degradation in acidic than alkaline, and in both cases degradation begun with the cracking of strongly hydrogen bonded urethane bonds and the destruction of ester bonds that originatedfrom PCL. The S-TPU(PCL)0.9 was characterized by the satisfactory biocompatibility with respect to C2C12 cells in comparison to the S-TPU(PCL)1.1, thus it may be suggested for further development in the medical field. When selecting the appropriate extrusion parameters, it is possible to obtain a stable filament. However, it is the production of small sections of the filament. In order to be able to receive the filament in a larger scale, the melt-extrusion system should be complemented with at least a cooling system (with a cooling tub), a laser measure of the diameter, and a system of the filament winding.

As a result of the studies, a 3D printing filament with well-characterized properties was obtained, which in the forthcoming tests will be evaluated for the printability of 3D structures while using the FDM 3D printer.

## Figures and Tables

**Table 1:** Review of medical-grade polyurethane systems in which polycaprolactone (PCL) is used as a polyol part of the chain.

Polyurethane System	Short Description	Year	Reference
(SF/LDI/PCLdiol/BDA) Silk fibroin poly(ester-urethane) urea	Tissue scaffolds with the structure of nanofibers for the regeneration of the heart valves obtained via electrospinning.	2018	[18]
(HDI/PCLtriol/PEG/glycerol) Crosslinked aliphatic poly(ester urethane)	Biodegradable polyurethane films with cross-linked hydrolysable bonds and a homogeneous structure for biomedical applications. PU with hydrogel behavior and susceptibility to hydrolytic degradation.	2015	[19]
(LDI-ε-caprolactone)block/LDI) Polyurethane block copolymer	Biodegradable PU with potential application in soft tissue engineering. A synthesis of a poly (L-lactide-ε-caprolactone) block copolymer was carried out, which was then used to react with L-lysine diisocyanate(LDI). The PU obtained can be used as a viscous injection which is cured in situ.	2017	[20]
(BDI/PCLdiol/L-Lysine ethyl ester dihydrochloride) Poly(ester-urethane)	Poly(ester-urethane) tissue scaffolds were obtained using the melt-extrusion additive manufacturing technique. The obtained scaffolds were cytocompatible and tested for use in the regeneration of myocardial tissue.	2014	[21]
(HDI/PCLdiol/BDO/Fe_2_O_3_ nanoparticles) Magnetic poly(ester-urethane) nanocmposite	A poly(ester-urethane) material of potential application for the regeneration of nerve tissue was obtained. The addition of nanoparticles improved the electrical conductivity, hydrophilicity and roughness of the obtained material. Biological tests show that nanocomposite was biocompatible and has suitable cell viability (in vitro cytotoxicity).	2014	[22]
(HDI/PCLdiol/PEG) Aliphatic poly(ester-urethane)	Electrospunednanofiberpoly(ester-urethane) membranes dedicated for guided bone regeneration. Obtained membranes had mechanical properties slightly higher than commercially available collagen or PTFE membrane.	2018	[23]

**Table 2:** Description of raw materials used in polyurethane synthesis.

Compound	Supplier	Short Description	Structure Formula
PCL diol (Capa^TM^ 2200)	Perstrop, Malmo, Sweden	Linear polyestrodiol, terminated with hydroxyl groups; Appearance: white, waxy solid; Average molecular weight: 2000 g/mol; Melting temp: 40–50 °C; Density: 1.05 g/cm^3^; Viscosity at 60 °C: 480 mPa s; Purity> 99%.	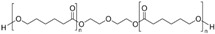
HDI	Sigma- -Aldrich, Taufkirchen, Germany	Aliphatic diisocyanate; Appearance: colorless liquid; Molar mass = 168.2 g/mol; Boiling point: 255 °C; Melting point: −67 °C; Density (25 °C) = 1.05 g/cm^3^; Purity> 99%; LD50 (rat) = 746 mg/kg.	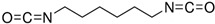
BDO	Brenntag, Essen, Germany	Low molecular weight chain extender Molar mass = 90.12 g/mol; Appearance: colorless liquid; Purity> 95.5%; Melting point: 20.4 °C; Density (20 °C) = 1.02 g/cm^3^	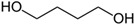

**Table 3:** Description of vibrations in FTIR spectra of S-TPU(PCL)0.9/1.1.

Wavelength [cm^−1^]	Band	Description
3330–3318w	*ν*NH	N-H stretching of urethane bond. Free and hydrogen bonded NH.
2917w, 2850w	*ν*CH_2_	Asymmetric and symmetric stretching C-H vibrations occurring in the aliphatic chains.
1717s	*ν*C=O	Stretching vibration of carbonyl group of PCL part.
1686vs, 1660w	*ν*C=O	Stretching vibration of carbonyl group occurring in the urethane bond; non-hydrogen bonded and strongly hydrogen bonded urethane group.
1542m	*δ*NH	N-H deformation of urethane bond (bending vibration).
1464m	*δ*CH_2_	C-H deformation (scissoring in plane).
1223s	*ν*N–C	Stretching vibration (urethane bonding).
1160s	*ν*C–O	Stretching vibration of ester (PCL part).
1065m, 1038m	*ν*C–O	Stretching vibration of C-O occurring in the urethane bond.
730v	γC–C	Skeletal vibrations of alkaline carbon chain (-C-C_n_-, n>4) present in HDI/ or PCL structure.
640m	*δ*N–H	Wide spectrum of N-H wagging, out of plane.

vw—very weak, w—weak, m—medium, s—strong, vs—very strong, v—variable.

**Table 4:** Thermogravimetric analysis (TGA) and DTG results for obtained polyurethanes.

Sample	TS^a^ (°C)	T_max_^b^(°C)		T_5%_^c^(°C)	T_30%_^d^(°C)	T_50%_^e^(°C)	T_offset_^f^(°C)
		I	II				
S-TPU(PCL)0.9	~260	351	430.6	283.8	330.3	344.8	455
S-TPU(PCL)1.1	~275	393.7	445	307.5	358.6	381.5	493

^a^ Thermal stability (up to 1% mass loss temperature), ^b^ First I/second II stage maximum rate of degradation temperature, ^c,d,e^5,30,50% mass loss temperature, ^f^ complete degradation temperature.

**Table 5:** Hardness, density, and tensile properties of synthesized materials.

Material Properties		S-TPU(PCL)0.9	S-TPU(PCL)1.1
Shore Hardness [°Sh]	A	84.36 ± 1.12	91.05 ± 4.86
	D	30.30 ± 1.27	36.97 ± 6.21
Density [g/cm^3^]		1.118 ± 0.007	1.021 ± 0.029
T_SB_ [MPa]		8.55 ± 0.49	21.40 ± 3.26
ε_b_[%] HS[%]		204.85 ± 13.74 29	726.32 ± 58.55 28

HS—theoretical content of hard segments.

**Table 6:** Contact angle measurements of S-TPU(PCL)0.9/1.1 with respect to various liquids.

Sample	Contact Angle Measurements			
	Diiodomethane	Formamide	Water	Ethylene Glycol
	[°]	[°]	[°]	[°]
S-TPU(PCL)0.9	63.22 ± 1.08	88.91 ± 2.08	104.43 ± 2.07	81.62 ± 0.82
	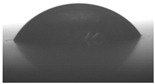	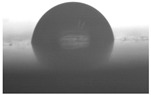	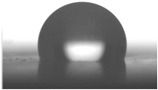	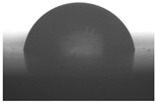
S-TPU(PCL)1.1	59.86 ± 1.28	90.10 ± 1.99	107.86 ± 0.88	83.15 ± 2.73
	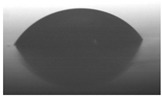	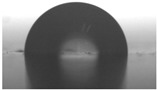	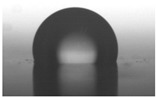	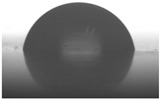

**Table 7:** Surface energy of S-TPU(PCL)0.9/1.1 calculated using the Owens–Wendt and acid-base method.

Sample	Owens-Wendt Method			Acid-Base Method				
	Total Surface Energy	Diperse Part	Polar Part	Total Surface Energy	L-W Part	Acid-Base Part	Acid Part	Base Part
	mN/m	mN/m	mN/m	mN/m	mN/m	mN/m	mN/m	mN/m
S-TPU(PCL)0.9	25.87	25.87	0.00	28.69	28.14	0.55	0.24	0.31
S-TPU(PCL)1.1	23.52	23.30	0.21	27.68	26.46	1.21	0.27	1.35

**Table 8:** Mass change of S-TPU(PCL) samples 0.9/1.1 during incubation in PBS at 37 °C.

Sample	Time of Incubation [Weeks]		
	1	4	12
	Mass Change [%]		
S-TPU(PCL)0.9	99.575 ± 0.062	99.887 ± 0.080	100.168 ± 0.069
S-TPU(PCL)1.1	99.972± 0.039	99.973 ± 0.039	100.182 ± 0.075

**Table 9:** Melt-extrusion parameters of F-TPU(PCL)0.9 * filament formation.

Process	T1 [°C]	T2 [°C]	Rotation Speed [rpm]	Dose Rate (g/min)	Filament Appearance
1	165	175	40	50	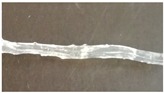
2	185	200	80	50	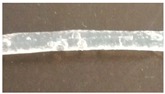
3	185	190	80	50	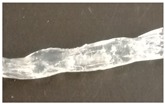
4	175	185	50	30	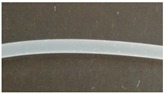

* For the filament formation stage the S-TPU(PCL)0.9 was selected due to, cytotoxicity results, higher swelling ratio, as well as lower water contact angle than S-TPU(PCL)1.1 sample.
